# Reimagining Cirrhosis Care for Older Adults: A 5Ms Approach

**DOI:** 10.3390/jcm15208186

**Published:** 2026-10-21

**Authors:** Manisha Verma, Lisa Deng, Tamar Taddei, Jennifer Lai

**Affiliations:** 1 Department of Medicine, Jefferson Einstein Philadelphia Hospital, Thomas Jefferson University, Philadelphia, PA 19141, USA; 2 Center for Data to Discovery and Delivery Innovation (3DI), San Francisco Veterans Affairs Health Care System, San Francisco, CA 94121, USA; 3 Department of Medicine, University of California, San Francisco, San Francisco, CA 94143, USA; 4 Division of Gastroenterology, Yale School of Medicine, New Haven, CT 06510, USA

**Keywords:** cirrhosis, age-friendly health systems, hepatology

## Abstract

**Background**: Cirrhosis is increasingly prevalent among older adults, driven in part by the rising burden of metabolic dysfunction-associated steatotic liver disease, which disproportionately affects this population. These patients are particularly vulnerable to premature geriatric syndromes, including frailty, disability, and cognitive impairment, reflecting the combined—if not synergistic—effects of aging and end-stage liver disease. As a result, they experience high symptom burden and health care utilization. Despite this complexity, cirrhosis care remains largely organized around disease-specific management and liver transplant evaluation, often failing to address geriatric and patient-centered needs. **Approach:** The Age-Friendly Health Systems initiative, developed by the John A. Hartford Foundation and the Institute for Healthcare Improvement, provides a standardized framework for high-quality care of older adults through the 4Ms: What Matters, Medication, Mentation, and Mobility. Although widely implemented across health systems, its application to hepatology remains limited. We propose an adaptation of the 4Ms framework for hepatology practice, supplemented by a fifth domain—Multicomplexity—to capture the intersecting challenges of symptom burden, multimorbidity, psychosocial vulnerability, and fragmented care. We describe how each domain can be operationalized within routine hepatology care and aligned with existing liver-specific tools and workflows. “What Matters” addresses prognostic uncertainty in cirrhosis and emphasizes structured advance care planning and goals-of-care conversations, particularly in the context of evolving transplant eligibility. “Medication” focuses on reducing polypharmacy and medication-related harm through careful prescribing in the setting of hepatic dysfunction and aging. “Mentation” targets identification and management of cognitive impairment, including minimal hepatic encephalopathy, alongside psychiatric comorbidities. “Mobility” emphasizes assessment and treatment of sarcopenia and frailty using liver-specific tools such as the Liver Frailty Index. “Multicomplexity” captures broader biopsychosocial and system-level challenges, including symptom burden, multimorbidity, social determinants of health, and care fragmentation. We further highlight palliative care as a complementary strategy for operationalizing the 5Ms in hepatology, supported by the PAL-LIVER trial, a multisite cluster randomized study demonstrating that trained hepatologist-delivered palliative care is noninferior to specialist-delivered care in improving quality of life and enhanced emotional well-being and patient satisfaction. **Conclusions:** An Age-Friendly, 5Ms-based approach adapted for hepatology has the potential to improve quality of life, reduce unnecessary health care utilization, and better align care with what matters most to older adults with cirrhosis. Integrating this framework into routine hepatology practice, supported by palliative care training principles and liver-specific tools, represents a scalable strategy for improving care delivery. Pragmatic implementation studies are needed to evaluate effectiveness, scalability, and sustainability across diverse health system settings.

## 1. Background

The epidemiology of cirrhosis is shifting toward older adults, reshaping hepatology practice and exposing gaps in traditional models of liver care. Adults aged 65 and older are now the fastest growing demographic among individuals with newly diagnosed cirrhosis [1,2]. This shift reflects the convergence of several trends. Metabolic dysfunction-associated steatotic liver disease (MASLD), now the leading cause of cirrhosis, is increasing in parallel with rising rates of obesity and type 2 diabetes [3]. Broader screening and noninvasive fibrosis assessment tools have contributed to expanded case identification [4]. Concurrently, population aging and increasing life expectancy provide a longer window for chronic liver diseases, including MASLD, to advance to cirrhosis.

As cirrhosis and aging intersect, patients experience compounded vulnerabilities that extend beyond liver-related complications. The aging liver undergoes significant physiologic changes, including reduced hepatic volume and diminished regenerative capacity, resulting in impaired drug metabolism and increased susceptibility to pathologic changes [5]. Polypharmacy is common in this population, and high-risk medications may precipitate or worsen hepatic encephalopathy, which can be difficult to distinguish from cognitive impairment and other neuropsychiatric disorders prevalent in older adults [6,7,8]. Cirrhosis-related therapies (e.g., diuretics, lactulose, non-selective beta blockers) may exacerbate geriatric syndromes. Concurrently, age-related sarcopenia is accelerated by altered catabolism in cirrhosis, increasing the risk for frailty and falls [9]. Despite substantial multimorbidity and poor prognosis associated with decompensated cirrhosis, advance care planning (ACP) is often inadequate in this population, contributing to high utilization of life-sustaining treatments at the end of life [10,11]. As a result, older adults with cirrhosis experience increased health care utilization, functional decline, disability, and mortality compared with age-equivalent adults [12]. Yet, cirrhosis care remains largely focused on managing liver-related complications (e.g., ascites, variceal bleeding, and hepatic encephalopathy) and transplant evaluation, often failing to address the multidimensional needs and priorities of older adults with cirrhosis.

The Age-Friendly Health Systems (AFHS) initiative, led by the John A. Hartford Foundation and the Institute for Healthcare Improvement (IHI), provides an evidence-based, practical framework for improving quality care for older adults through the 4Ms: What Matters, Medication, Mentation, and Mobility. AFHS implementation has demonstrated measurable impact: reduced hospital readmissions, deprescribing of inappropriate medications, and increased rates of ACP [13,14,15,16]. While over 7000 care settings have implemented the 4Ms, its applicability to hepatology is unknown, with only one IHI-recognized hepatology setting (San Francisco Veterans Affairs Liver Clinic) and no published implementation studies to date [17]. This gap represents an important opportunity to integrate geriatric principles into routine liver care. Hepatologists are uniquely positioned to implement the 4Ms because each domain aligns closely with common challenges encountered in cirrhosis care, and validated liver-specific tools already exist to support assessment and intervention within this framework.

While the AFHS initiative provides evidence-based tools and best practices to assess and act on the 4Ms in older adults, the existing 4Ms framework does not include liver-specific considerations. In this article, we highlight the unique needs of older adults with cirrhosis and present practical strategies to incorporate the 4Ms and a fifth M—Multicomplexity—into hepatology care (Table 1) [18]. We also describe how findings from the recent PAL-LIVER trial, a large cluster randomized trial comparing palliative care (PC) delivered by PC specialists to that delivered by trained hepatologists, complement the 5Ms-based approach to care delivery in older adults with cirrhosis [19]. Although AFHS was primarily designed for adults aged 65 years and older, its application may also extend to younger patients with cirrhosis, in whom accelerated aging can result in phenotypes similar to those observed in older adults. Together, these approaches can help align hepatology care with the priorities and needs of older adults across the spectrum of cirrhosis, irrespective of liver transplant eligibility, with the goal of improving quality of care and patient outcomes (Table 2).

Table 1 provides a list of evidence-based assessment tools in the Age-Friendly Health Systems toolkit and suggested tools for the cirrhosis population as denoted by asterisk (*). We suggest adding QuickStroop or PHES to screen for minimal hepatic encephalopathy, Liver Frailty Index to assess for frailty, LiverTox database to identify medication-related hepatotoxicity, Edmonton Symptom Assessment System to assess for symptoms, Hunger Vital Sign to screen for food insecurity, and Zarit Burden Interview to screen for caregiver burden. Tools were selected based on relevance and/or evidence in liver disease and ease of implementation. Selection of assessment tools, responsible team members, and corresponding management strategies should be individualized to the local care setting.

## 2. Applying the 5Ms Framework to the Unique Needs of Older Adults with Cirrhosis

### 2.1. Competing Goals and Prognostic Uncertainty (What Matters)

Older adults with cirrhosis face an unpredictable disease trajectory characterized by periods of stability punctuated by acute decompensation. As a result, they experience disproportionately high health care utilization near the end-of-life compared with age-equivalent adults, yet ACP remains infrequent, particularly among patients awaiting liver transplantation [11]. The uncertainty surrounding prognosis, treatment burden, and transplant eligibility underscores the importance of aligning care with patients’ values, priorities, and evolving goals.

Hepatologists are uniquely positioned to facilitate goals of care conversations. Their expertise in liver-specific prognostication and liver transplant candidacy informs decisions regarding management of cirrhosis-related complications, such as procedures (e.g., paracentesis, endoscopy), hospitalizations, and end-of-life care. The PAL-LIVER trial, a multisite cluster randomized comparative effectiveness trial conducted across 19 U.S. medical centers, compared PC delivered by trained hepatologists with that delivered by PC specialists. Hepatologists completed a 12-week virtual training focused on core PC skills, communication and goals of care conversations, symptom management, and psychosocial support in the context of end stage liver disease. The trial showed non-inferiority of hepatologist delivered PC in improving quality of life of patients with cirrhosis at 3 months, with better improvement in emotional well-being and patient satisfaction, as compared with PC specialist delivered care [19]. These findings support the fact that hepatologists can deliver supportive care as a part of their routine practice, with additional training.

Early integration of PC, either through trained hepatologists or specialty PC co-management, can ensure that care plans reflect patient values, particularly in the context of limited transplant eligibility. PC integration can also promote timely ACP discussions and appropriate use of hospice services. ACP discussions should ideally occur early in the disease course and prior to the onset of hepatic encephalopathy or cognitive impairment. It is particularly important in patients with cirrhosis to identify and document a surrogate decision-maker in an advance directive, such as durable power of attorney for health care and/or living will [20]. Clinicians should prepare surrogate decision-makers by promoting a shared understanding of the patient’s values and goals, prognosis, and preferences for care should the patient become incapacitated [21].

“What Matters” should be elicited as an ongoing, patient-centered process rather than a single formal intervention. While structured interventions such as PAL-LIVER demonstrate the potential benefit of training hepatology clinicians in serious illness communication, similar principles can be incorporated into routine care using brief, structured conversations focused on patients’ values, goals, understanding of illness, treatment preferences, and priorities for quality of life. These conversations should be revisited as cirrhosis progresses and clinical circumstances change, with involvement of specialty PC when additional support is needed. Importantly, eliciting “What Matters” is an interdisciplinary responsibility; nurses, social workers, advanced practice providers, physicians, chaplains, and other members of the care team may identify opportunities to explore patient and caregiver priorities, facilitate communication, and ensure that these preferences are incorporated into the overall care plan. This team-based approach is consistent with recommendations for integrating palliative and supportive care into the management of advanced cirrhosis.

Practical implementation strategies include training hepatologists on effective serious illness communication, incorporating standardized ACP documentation templates into the electronic health record, providing patient-facing ACP resources, and routinely revisiting goals following major clinical transitions such as hospitalizations, hepatic decompensation and liver transplant de-listing.

### 2.2. Polypharmacy in Liver Dysfunction (Medication)

Medication management is particularly challenging in older adults with cirrhosis. Hepatic dysfunction, compounded by age-related changes in drug metabolism, increases susceptibility to medication-related adverse events. Yet, potentially inappropriate and high-risk medications, such as benzodiazepines, opioids, and nonsteroidal anti-inflammatory drugs, are commonly used among patients with cirrhosis [6]. Polypharmacy is highly prevalent; patients with cirrhosis take a median of nine medications [7]. Over-the-counter medications (e.g., acetaminophen) and herbal supplements pose additional risks for drug-induced liver injury, particularly among older adults with cirrhosis.

Hepatologists can leverage their expertise to optimize medication use by balancing therapeutic benefits against risks related to hepatic dysfunction, frailty, and comorbidities. The Beers Criteria is an expert consensus-derived list of medications commonly used to identify inappropriate medication use in older adults [22]. However, the Beers Criteria do not fully capture the medication-related risks unique to cirrhosis. Liver-specific resources, such as LiverTox, can complement geriatric prescribing guidance to inform medication safety, dose adjustment, and deprescribing decisions [22,23]. General principles for pharmacotherapy in older adults with cirrhosis include initiating medications at low doses with slow titration and cautious monitoring for adverse effects. Time limited trials with regular reassessments can help determine ongoing benefit of medications.

Cirrhosis-specific therapies should similarly be individualized to the older adult’s comorbidities, functional status, and vulnerability to adverse effects. For example, non-selective beta blockers and diuretics have been associated with increased fall risk among older adults [24]. These medications are not contraindicated in older adults with cirrhosis, but their benefits should be weighed against potential risks, particularly in patients at increased risk of falls, and require close monitoring. Lactulose therapy for hepatic encephalopathy may pose additional challenges in older adults, including dehydration, mobility limitations, and incontinence. Rifaximin monotherapy should be considered for patients who cannot tolerate lactulose. Further research is needed to identify common medication-related problems in older adults with cirrhosis and to characterize the risks and benefits of cirrhosis-specific therapies in this population.

Practical approaches include pharmacist-led medication reconciliation of both prescribed and over-the-counter medications, use of prescribing checklists tailored to cirrhosis, and clinical decision support tools that flag high-risk medications or supplements. Nonpharmacologic approaches for common symptoms such as pain and muscle cramps should also be incorporated when appropriate [25].

### 2.3. Hepatic Encephalopathy, Cognitive Impairment, and Psychiatric Comorbidities (Mentation)

Cognitive dysfunction in older adults with cirrhosis presents a diagnostic conundrum. Hepatic encephalopathy, dementia, and depression frequently co-exist and may be difficult to distinguish clinically. Minimal hepatic encephalopathy, the earliest and subclinical stage of hepatic encephalopathy (HE), affects up to 80% of patients with cirrhosis and is challenging to diagnose without specialized tests [26]. Yet, these subtle cognitive deficits may impair medication adherence, self-management, and decision-making capacity, and are associated with falls and decreased quality of life [27].

For the Mentation domain, the AFHS 4Ms framework includes Mini-Cog [28] and Montreal Cognitive Assessment (MoCA) [29] to screen for cognitive impairment and the Patient Health Questionnaire (PHQ-2 or PHQ-9) [30,31] to screen for depression. We advocate that minimal hepatic encephalopathy screening is a critical part of cognitive assessment in older adults with cirrhosis, for whom subtle impairments can have profound detrimental effects and these impairments can be treated with medications such as lactulose or rifaximin. We recommend adding validated hepatic encephalopathy diagnostic tools such as the QuickStroop or Psychometric Hepatic Encephalopathy Score to the 5Ms framework for older adults with cirrhosis [32,33]. If minimal hepatic encephalopathy is suspected, a time limited trial of lactulose should be considered with repeat cognitive testing at follow up.

Notably, hepatic encephalopathy is a key determinant of caregiver burden among patients with cirrhosis [34]. A PC approach is especially valuable in helping patients and caregivers cope with hepatic encephalopathy and cognitive impairment through targeted education, anticipatory guidance and shared decision-making. Early recognition of cognitive changes can enhance management and reduce preventable hospitalizations.

### 2.4. Cirrhosis-Driven Sarcopenia and Frailty (Mobility)

Age-related sarcopenia is accelerated by cirrhosis-related catabolic state, systemic inflammation, and malnutrition. While the AFHS 4Ms framework incorporates validated mobility assessments in older adults such as the Timed Up and Go [35] and Tinetti Performance Oriented Mobility Assessment [36], widely established liver-specific frailty assessments are not included. Frailty as assessed by the Liver Frailty Index, a validated liver-specific measure, is independently associated with disability, mortality, hospitalizations, and post-liver transplant outcomes in patients with cirrhosis [37,38]. Embedding liver-specific frailty assessment into the Mobility domain would not only better capture frailty and associated risks in patients with cirrhosis but also complement existing hepatology and liver transplant care.

Recognition of frailty has important management implications, as interventions targeting nutrition, complications and physical function can contribute to improvement in patient well-being. However, there are several unique management considerations in patients with cirrhosis. Due to increased muscle protein catabolism and “accelerated starvation” among patients with cirrhosis, evidence supports benefits of a high-protein diet (1.2–1.5 g/kg/day) and small frequent meals with nighttime nutrition supplement. Vitamin D deficiency and reduced bone mineral density frequently occur in patients with liver disease—routine screening and supplementation should be provided as appropriate. Liver-specific complications that affect mobility should also be optimized, such as hepatic encephalopathy or ascites.

Exercise interventions, including resistance and aerobic training, can improve muscle mass and function and may provide additional liver-specific benefits in patients with MASLD. As more older adults are being considered for liver transplantation, there are emerging data that prehabilitation programs may improve physical function, waitlist survival, and decreased post-transplant length of stay [39].

### 2.5. Symptom Burden, Multimorbidity and Care Fragmentation (Multicomplexity)

Older adults with cirrhosis often experience multiple concurrent symptoms, with prevalence comparable to other advanced diseases such as cancer and heart failure [40]. Symptom burden not only contributes to poor quality of life but is also associated with frailty and high health care utilization [41]. Comorbidity is common and can further complicate management due to potentially competing priorities, polypharmacy and fragmented care plans.

Beyond medical complexity, older adults with cirrhosis also have substantial biopsychosocial needs. Social determinants of health, such as low income, food insecurity, and social isolation, are strongly associated with cirrhosis and hepatocellular carcinoma-related outcomes [42]. In addition, patients often receive care across multiple settings, including specialty clinics, emergency departments, and inpatient units increasing the risk of fragmentation. Together, symptom burden, multimorbidity, psychosocial vulnerability, and care fragmentation represent the broader concept of “Multicomplexity” in cirrhosis.

Implementation strategies should focus on identifying and addressing Multicomplexity across the 4Ms domains. Tools such as the Edmonton Symptom Assessment System [43] can be used to assess symptom burden, Hunger Vital Sign [44] to screen for food insecurity, and Zarit Burden Interview [45] to screen for caregiver burden. PC, delivered by trained hepatologists or specialty PC, is particularly well repositioned to address Multicomplexity through:

- *Symptom Management*: PC provides structured approaches to managing common symptoms in older adults with cirrhosis such as pain, dyspnea, pruritus, and fatigue. These symptoms are frequently under-treated in cirrhosis and significantly affect quality of life.
- *Psychosocial Support*: The interdisciplinary palliative care team is well positioned to address social, emotional, spiritual and caregiver needs that may extend beyond the resources available in routine hepatology care and promote equitable delivery of 5Ms framework.
- *Care Coordination and Transitions*: By improving communication across settings and focusing on continuity, PC can reduce unnecessary hospitalizations and improve patient and caregiver experience.

As workforce shortages and increasing patient complexity challenge health systems, this model offers a scalable approach for integrating person-centered care into specialty practice [46].

## 3. Implementation and Evaluation Considerations

**Figure 1:**
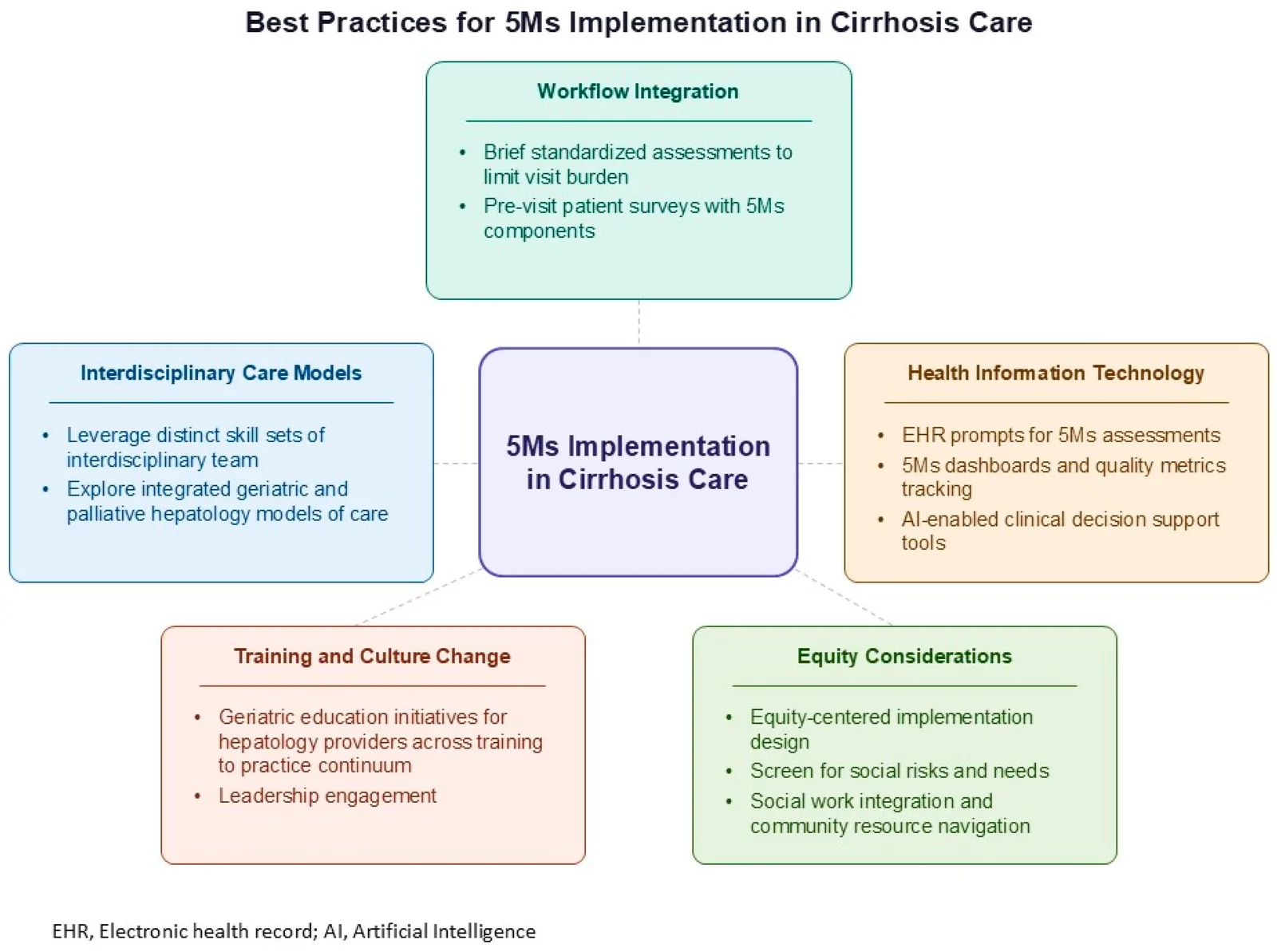
Best practices for 5Ms Implementation in Cirrhosis Care.

Successful implementation of the 5Ms framework in hepatology requires a multifaceted approach that includes workflow integration, interdisciplinary care models, provider education, health information technology support, and attention to health equity (Figure 1). Implementation evaluation should also adopt a multidimensional approach and include both clinical and implementation outcomes (Table 3). Implementation science frameworks, such as RE-AIM (Reach, Effectiveness, Adoption, Implementation, Maintenance), can guide evaluation and inform scalability [47].

Delivering age-friendly cirrhosis care is challenged by a limited workforce in geriatrics, palliative care, and hepatology. Given these shortages, additional training of hepatologists, and interdisciplinary collaboration among physicians, advanced practice providers (APPs), and other healthcare professionals is essential to effectively implement the 5Ms framework and meet the complex needs of older adults with cirrhosis.

## 4. Conclusions and Future Directions

Older adults with cirrhosis have complex, multidimensional needs that are not fully addressed by traditional disease-focused models of care. The rapid aging of the cirrhosis population, combined with increasing clinical complexity, underscores the need to redesign hepatology care around age-friendly, person-centered principles. Integrating the 5Ms into hepatology practice—particularly when combined with palliative care—has the potential to improve quality of life, reduce unnecessary health care utilization, and ensure that care aligns with what matters most to older adults with cirrhosis.

Further research is needed to evaluate the effectiveness of the 5Ms framework in cirrhosis-specific populations. Priorities include:

- Developing and validating cirrhosis-specific tools aligned with the 5Ms, such as measuring goal concordant care (recently validated as a part of PAL-LIVER trial) [48].
- Testing integrated care models that combine hepatology, geriatrics, and palliative care [49]
- Evaluating cost-effectiveness and impact on healthcare utilization
- Identifying scalable strategies to improve adoption in diverse hepatology care settings

Pragmatic trials and real-world implementation studies will be essential to translate age-friendly principles into routine hepatology practice.

## Figures and Tables

**Table 1:** Practical implementation of age-friendly cirrhosis care in ambulatory hepatology.

Domain	Assessment Tools	Interdisciplinary Team	Next Steps
**What Matters**	One Question Screen example: How could we help you in achieving your goals? PREPARE for Your Care POLST Advance Directive	Any clinical team member: MD/APP RN MA Social Worker Chaplain	Align treatment plan with goals of care Revisit at each major clinical transition (e.g., hospitalization, disease progression, liver transplant de-listing)
**Mentation**	*Cognitive impairment:* Mini-Cog MoCA *Depression* PHQ-2 PHQ-9 *Hepatic encephalopathy* QuickStroop (1–2 min) * Psychometric Hepatic Encephalopathy Score (PHES) (5–15 min) *	RN or MA administers MD/APP interprets Social Worker	Referral to neurology for cognitive impairment evaluation Referral to behavioral health services For suspected HE, review potential confounders that could contribute to cognitive impairment Time limited trial of lactulose for suspected HE and reevaluate diagnosis if no response. Rifaximin monotherapy for older adults who cannot tolerate lactulose
**Mobility**	Timed Up and Go (TUG) Tinetti Performance Oriented Mobility Assessment (POMA) *Liver-specific:* Liver Frailty Index (1–3 min) *	RN or MA administers MD/APP Dietitian	Referral to physical therapy Referral to occupational therapy Referral to dietitian for liver-tailored nutrition optimization Consider other exercise interventions based on patient preference (e.g., yoga, tai chi) Address liver-disease factors and complications contributing to impairments
**Medication**	Beers Criteria *Liver-specific:* LiverTox Database * Over the counter medications Herbal supplements	Pharmacist MD/APP RN	Multidisciplinary collaboration to re-evaluate treatment risk Reduce dose of medications or de-prescribe Offer nonpharmacologic treatment options
**Multicomplexity**	Edmonton Symptom Assessment System * Hunger Vital Sign (2-item) * Zarit Burden Interview (abbreviated 1 or 4 items) *	RN or MA administers MD/APP Social Worker Chaplain	Hepatologist-delivered palliative care or specialty palliative care referral Social work referral for assessment and connection to community resources

MD/APP, physician or advanced practice provider; POLST, Physician Orders for Life-Sustaining Treatment; MoCA, Montreal Cognitive Assessment; PHQ-2, Patient Health Questionnaire—2; PHQ-9, Patient Health Questionnaire—9; HE, hepatic encephalopathy; MA, medical assistant; RN, registered nurse.

**Table 2:** Applying the 5Ms framework across the spectrum of cirrhosis stages in older adults.

Disease Stage	Main Concerns	Priority 5Ms	Examples of Interventions
Compensated cirrhosis	Preservation of function, prevention of decompensation, comorbidity burden, long-term goals	What Matters, Mobility, Medication, Multicomplexity, Mentation	Assess function and mobility; optimize medications and comorbidities; address preventive care and modifiable risks; establish patient priorities and goals; screen for cognitive concerns when clinically indicated
Early decompensated cirrhosis	Ascites, hepatic encephalopathy, variceal bleeding, nutritional decline, increasing symptom burden	Mentation, Medication, Mobility, Multicomplexity, What Matters	Manage cirrhosis complications; review medications and potentially inappropriate medications; assess frailty/sarcopenia and mobility; address nutritional and functional needs; incorporate patient goals and caregiver needs
Recurrent/further decompensation	Recurrent complications and hospitalization, frailty, sarcopenia, symptom burden, declining function	What Matters, Mentation, Mobility, Medication, Multicomplexity	Multidisciplinary management; prevent recurrent complications; address frailty, nutrition and function; optimize medications; assess caregiver burden and treatment preferences; consider transplant and/or palliative care according to goals
Advanced disease/transplant evaluation	Transplant candidacy, frailty, comorbidities, cognitive and functional status, treatment burden, goals of care	What Matters, Mobility, Mentation, Multicomplexity, Medication	Comprehensive transplant assessment; optimize nutrition, mobility and frailty; evaluate cognition and psychosocial support; review medication burden; assess comorbidities; use shared decision-making to align treatment with patient goals

**Table 3:** Measuring impact of Age-Friendly Cirrhosis Care.

Domain	Outcome Measure
Patient-centered outcomes	Quality of life, symptom burden, goal concordance
Clinical outcomes	Hospitalizations, readmissions, emergency department visits
Process outcomes	Documentation of goals-of-care discussions, cognitive screening rates, medication reconciliation rates
Functional outcomes	Mobility, frailty indices, independence in activities of daily living
Caregiver outcomes	Burden, satisfaction, preparedness

## Data Availability

No new data were created or analyzed in this study. Data sharing is not applicable to this article.
